# Integrated Newborn Screening in Nigeria: The Way Forward, A Workshop Report

**DOI:** 10.3390/ijns12010005

**Published:** 2026-01-29

**Authors:** Olumuyiwa S. Folayan, Bose E. Orimadegun, Adejumoke I. Ayede, Baba P. Inusa, Marika K. Kase, John I. Anetor

**Affiliations:** 1 Institute of Cardiovascular Disease, University of Ibadan, Ibadan 200212, Nigeria; olumuyiwa.folayan@npmcn.edu.ng; 2 Department of Paediatrics, University of Ibadan/University College Hospital, Ibadan 200212, Nigeria; idayede@yahoo.co.uk; 3 Department of Chemical Pathology, University of Ibadan, Ibadan 200212, Nigeria; orimadegunbose@yahoo.co.uk; 4 Department of Chemical Pathology, University College Hospital, Ibadan 200212, Nigeria; 5 Centre for African Newborn Health and Nutrition, University College Hospital, Ibadan 200212, Nigeria; 6 Faculty of Life Sciences & Medicine, Kings College London, London WC2R 2LS, UK; baba.inusa@gstt.nhs.net; 7 Strategic Initiatives Reproductive Health, Revvity, Inc., PL 10, 10101 Turku, Finland; marika.kase@revvity.com

**Keywords:** integrated newborn screening, sickle cell disease, congenital hypothyroidism, public health, policy formulation

## Abstract

Newborn screening (NBS) is a cost-effective public health strategy for the early detection of congenital disorders that cause neonatal/infant morbidity and mortality. It is standard care in many high-income and emerging economies. Nigeria, despite its high birth number, has no newborn screening (NBS) programme for any disorder, causing missed opportunities for early therapy. This manuscript is a workshop report and expert consensus of a three-day national workshop organised by the Newborn Screening Consortium–Nigeria (NSC-N) in conjunction with The Federal Ministry of Health Nigeria, Revvity, and international partners. The first meeting comprised experts in different fields of newborn screening and newborn care who reviewed priority congenital disorders, implementation barriers, and national NBS needs in Nigeria. Experts presented pilot data, opinions, and global best practice evidence. Contributions were examined and debated and conclusions were reached by guided discussions and consensus agreement for a pragmatic nationwide NBS plan. The key outcomes were the urgency for Nigeria to begin an integrated, comprehensive NBS programme. Based on standard prioritisation criteria, sickle cell disease and congenital hypothyroidism were selected. Key implementation strategies included integration into routine maternal and child health services, establishing a national screening database, and developing a robust legislative and policy framework. The NBS workshop developed a framework to commence and incorporate integrated NBS into the Nigerian healthcare system. Two conditions were selected to kickstart the programme and establish a foundation for future expansion. This would improve neonatal health outcomes and reduce the long-term burden of congenital disorders.

## 1. Introduction

Newborn screening (NBS) has emerged as a cornerstone of early-life preventive health [1]. It enables the timely detection and treatment of congenital birth disorders, inherited metabolic disorders, and other conditions present from birth that would otherwise result in lifelong disability or premature death [1,2]. Newborn screening is not merely a blood test, but a transformative public health service that has been demonstrated to be extremely effective in reducing morbidity [1]. Globally, more than 60 conditions can be screened for [1,2,3]; these vary widely across countries depending on the available resources, level of development, available technology, and disease prevalence. The conditions that are commonly screened for range from haemoglobinopathies, phenylketonuria (PKU), cystinosis, maple syrup urine disease (MSU), and transport disorders such as cystic fibrosis (CF) among many Caucasian communities, and metabolic disorders like congenital hypothyroidism (CH) among other numerous conditions. The newborn screening programme in the Philippines that began with 5 conditions has expanded to a panel that screens at least 19 newborn conditions [4]. Nigeria, a nation with a burgeoning population and substantial healthcare challenges, has, however, not implemented any national newborn screening programme [1,5,6]. Current practices are fragmented and limited to pilot projects or isolated institutional efforts [1]. This gap has caused preventable disabilities, reduced life expectancy, and considerable socioeconomic hardships for many individuals [7]. Children with undetected conditions may also require specialised care that is difficult to access, perpetuating a cycle of inadequate healthcare delivery and increased financial burden on the family and society [7].

Recognising these challenges, the Newborn Screening Consortium–Nigeria (NSC-N) convened a national workshop in Lagos, Nigeria, from the 19th to the 21st of November, 2024, titled ‘Integrated Newborn Screening in Nigeria–The Way to Go’. The workshop was a collaboration between the Newborn screening Consortium–Nigeria (NSC-N) and Revvity. Other key participants included the International Federation of Clinical Chemistry and Laboratory Medicine (IFCC), the African Federation of Clinical Chemists (AFCC), the International Society for Neonatal Screening (ISNS), the Federal Ministry of Health, Nigeria (FMOH), the Partnership for Maternal, Newborn & Child Health (PMNCH), and the University of Ibadan. The collective expertise and collaborative dialogue at the workshop provided the impetus for comprehensive recommendations to integrate newborn screening into routine maternal and child health services across Nigeria. The main aim of this report is to document the outcome of the first national workshop on integrated newborn screening in Nigeria, and propose a strategic framework for newborn screening implementation in Nigeria and canvas government support.

### 1.1. Organisation

The selection of the workshop participants was largely based on the existing six health zones in Nigeria, the Federal Ministry of Health, and non-governmental organisations involved in maternal and child health. The workshop was attended by 54 people drawn from these organisations and was funded by Revvity. The workshop included experts in the fields of newborn screening and newborn care to review priority congenital disorders, implementation barriers, and national NBS needs in Nigeria. The selected experts presented pilot data, opinions, and evidence for global best practice. All contributions were examined and debated, and conclusions were reached by guided discussions and consensus agreement for a pragmatic, nationwide NBS plan.

### 1.2. Summary of Presentations

Opening remarks were made by the team lead, NSC-N, Professor John Anetor, stressing the pivotal public health significance of NBS and its growth since 1960s when it was started by Robert Guthrie, the ‘Father of NBS’. He urged Nigeria to start with a few disorders and then expand the scope.

### 1.3. Patient/Parent Voice

Deborah, a parent advocate, set the stage for the discussions at the workshop. She shared the life-changing experience of her family after their child’s diagnosis of sickle cell disease through newborn screening. Her account emphasised the need for early detection, family support, and consistent follow-up in improving the quality of life in children living with sickle cell disorder. She concluded by highlighting the importance of equitable and integrated newborn screening systems for advancing child survival and health equity in Africa.

### 1.4. Laboratory Perspectives

Professor John I. Anetor, a Chemical Pathologist at the University of Ibadan and the convener of the workshop, delivered a series of presentations outlining the scientific rationale, methods, and policy imperatives for newborn screening (NBS) in Nigeria. In his talk, he said that many inborn errors of metabolism are treatable when identified early. He also reviewed advanced diagnostic platforms, including high-performance liquid chromatography (HPLC), tandem mass spectrometry (MS/MS), and genomic sequencing. Although these advanced technologies are gaining some traction in newborn screening, current newborn screening programmes worldwide are largely based on conventional biochemical screening methods including immunoassay, colorimetry, fluorometry, and mass spectrometry [8]. Genomic screening is an emerging tool that is not widely employed in newborn screening programmes [8]. He stressed that the advanced methods are not limitations to starting a newborn screening programme, and may be an expansion plan after the programme in Nigeria is well established.

He proposed a tiered model suited to our local context. In his proposed model, NBS is initiated using simple biochemical assays in the primary setting, with positive cases referred to central reference laboratories. He concluded that a phased implementation and a firm policy commitment are essential for a sustainable national NBS programme.

### 1.5. Clinician Perspectives

Professor Ayede, a Consultant Neonatologist at University College Hospital, Ibadan, and co-team lead, addressed the need to institutionalise and scale up newborn screening systems nationwide. She noted that Sub-Saharan Africa contributes to nearly 30% of global congenital anomalies, but has weak integrated screening frameworks [9]. Her presentation called for strengthened institutional collaboration, robust policy formulation, and data-driven planning to reinforce Nigeria’s newborn health structure. She proposed that linking NBS with immunisation and neonatal intensive care would help to achieve coverage.

Dr Oluwakemi Ashubu, a Paediatric Endocrinologist at the University College Hospital, Ibadan, spoke about newborn screening for congenital hypothyroidism, a leading cause of preventable intellectual disability. She emphasised the critical window for screening within 72 h of life and initiation of levothyroxine therapy within two weeks for optimal neurodevelopmental outcomes [10,11]. Her talk advocated for the strengthening of national screening and follow-up systems to promote cognitive health in children.

### 1.6. Industry Perspectives

Marika Kase, Business Director for Strategic Initiatives in Reproductive Health and Newborn Screening at Revvity, examined global disparities in NBS coverage, particularly across low and middle-income countries (LMICs). She highlighted the cost-effectiveness and life-saving benefits of the early detection of congenital disorders. She also urged that Sub-Saharan Africa should prioritise screening for congenital hypothyroidism and sickle cell disease due to their high prevalence and ease of intervention. She called for global collaboration, leadership, and the establishment of sustainable infrastructure to bridge the equity gap in access to newborn screening.

### 1.7. Public Health Perspectives

Mr Jelili Ojodu, a Senior Director for Newborn Screening and Genetics at the Association of Public Health Laboratories (APHL), USA, described newborn screening as one of the most impactful newborn public health interventions. He presented the use of dried blood spot (DBS) technology as a cost-effective and scalable method suited to resource-limited settings. He also emphasised the importance of quality assurance, workforce training, and data-driven evaluation. He concluded his talk with an advocacy for global knowledge sharing and partnerships to strengthen and sustain NBS programmes.

Professor Baba Inusa, a Consultant Paediatric Haematologist at King’s College London, and National Chair of the UK Haemoglobinopathy Panel, presented the African Research and Innovative Initiative for Sickle Cell Education (ARISE). He detailed the network’s achievements in enhancing capacity across Africa through the training of over 300 health professionals, virtual education programmes reaching more than 2000 participants, and staff exchanges supporting laboratory and research. He also spoke on ARISE’s role in establishing external quality assurance systems and quality laboratory programmes in Nigeria, Zambia, and Angola.

Oyeyemi Temilade Pitan, a chartered accountant and public health advocate, presented the role of youth-led and community-driven advocacy in advancing universal health coverage and equity. She demonstrated how her work at the Gem Hub Initiative created interventions that addressed sexual and reproductive health rights, mental health, and youth empowerment in underserved communities. Her contribution highlighted the importance of civil society engagement in driving health reforms that can ensure community-owned newborn screening efforts.

### 1.8. Implementation Programme

Dr Livingstone Dogara shared insights from his career in haematology, focusing on translating laboratory research into clinical and community impact. He highlighted his ongoing work in capacity building, clinical trials, and implementation science aimed at strengthening comprehensive sickle cell care across Africa.

Dr Victoria Odesina, a veteran nurse researcher and co-founder of the Global Sickle Cell Alliance (GLOSCA), described her roles as a parent of two children living with sickle cell disease, and a public health nurse with strong advocacy qualities. She discussed GLOSCA’s contributions to developing newborn screening and follow-up programmes in Nigeria and beyond in her four decades of public health practice. The barriers she identified included limited diagnostic infrastructure, stigma, and inequity. She called for stronger public–private partnerships and investment to ensure equitable access to newborn and genetic screening.

### 1.9. International Best Practice

Professor Bonham, the President of the International Society of Neonatal Screening and Co-Chair of the IFCC/ISNS global Taskforce on Newborn Screening (TF-NBS), spoke on the global progress, challenges, and future directions of NBS. He reiterated the need for equitable access to early diagnosis and treatment for newborns in LMICs. He highlighted how strengthening laboratory capacity and international partnerships can bring about outcomes. His talk also identified the importance of sustainable public health frameworks, shared expertise, and data-driven policies in scaling up NBS programmes worldwide. These would ensure that every child, regardless of where they are born, would benefit from the early detection of inherited and metabolic disorders.

### 1.10. World Health Organisation

Dr Ayesha De Costa, representing the World Health Organisation, offered a global perspective on prioritising birth defect screening in low- and middle-income countries. She noted that 90% of children with serious congenital anomalies are born in LMICs [9], and advocated for integrating newborn screening into routine health systems. She emphasised that newborn screening should be viewed as a system encompassing biochemical, clinical, and functional assessments. She also observed that it should also incorporate screening for congenital heart and hearing defects alongside diagnosis, referral, and rehabilitation. She urged for the strengthening of health systems, improvement of surgical capacity, and improvement of governance structures to ensure the reduction in under-five mortality due to birth defects.

## 2. Discussion

### 2.1. Current Situation of Newborn Screening Gaps in Nigeria

1. NBS practice is not routine in Nigeria, unlike in many countries that have established programmes that screen up to 60 conditions, depending on available resources. The unavailability of a standard screening protocol in Nigeria means that many affected neonates remain undiagnosed.
2. Many opportunities for early intervention are lost because parents and healthcare providers are unaware of the potential long-term benefits of the early detection of congenital disorders.
3. The laboratory infrastructure, personnel, and protocols needed to investigate and interpret screening tests accurately are lacking in the country.
4. The current Nigerian national health budget does not include newborn screening, and the absence of a supportive legal and policy framework undermines efforts to mandate routine newborn screening.

### 2.2. Prioritisation of Target Conditions

Given the many candidate congenital conditions eligible for screening in Nigeria, the expert panel agreed that a phased approach would be most pragmatic. Two conditions were prioritised based on their prevalence, clinical impact, and the feasibility of early intervention. These are sickle cell disease (SCD) and congenital hypothyroidism (CH), which both meet the Wilson and Jungner criteria for population-based screening [12]. In Nigeria, both conditions contribute to significant early childhood morbidity and sickle to mortality, yet remain clinically silent at birth. Their natural histories are well-defined, screening tests are available, and early detection enables low-cost interventions that improve survival and neurodevelopmental outcomes. These support the epidemiological justification for integrating SCD and CH into national newborn screening strategies in Nigeria [12].

Nigeria’s recent birth rate estimates approximates 7.56 million live births per year [13,14], reflecting sustained demographic growth and a large newborn population at risk of early childhood disease burdens. The country’s crude birth rate remains elevated, at around 33 births per 1000 people per year, compared with a global average below 18 per 1000 [13]. A systematic review and meta-analysis reported a pooled prevalence of sickle cell disease (SCD) of approximately 4% (95% CI 3–6%) among Nigerian children and adolescents, with sickle trait carriers accounting for a significantly higher proportion of the population [15,16]. A 4% prevalence applied to Nigeria’s substantial annual birth cohort suggests that over 300,000 infants may be born with SCD each year, making sickle cell disease a public health burden.

Congenital hypothyroidism is a leading cause of preventable intellectual disability [10]. Early detection through newborn screening enables initiation of thyroid hormone replacement therapy within the first two weeks of life [10,11]. This averts the neurodevelopmental delay and ensures normal cognitive development [11]. The lack of comprehensive, population-based newborn screening programmes in Nigeria reinforces the need to prioritise CH detection alongside other conditions in national NBS efforts [3]. While national data on the incidence of congenital hypothyroidism (CH) in Nigeria remain limited due to the absence of systematic newborn screening, available hospital-based studies indicate that CH occurs at an appreciable frequency. Reported incidence rates range from 0.10% to 0.14% in different parts of the country [3,17]. When extrapolated to Nigeria’s large annual birth cohort [13,14], these estimates suggest that tens of thousands of infants may be affected each year, highlighting a substantial yet under-recognised public health burden.

Other rarer conditions in Nigeria, such as fatty acid oxidation disorders, organic acidemias, and other inborn errors of metabolism, were not included in the initial implementation stage of the national newborn screening programme. In addition, conditions such as phenylketonuria, galactosemia, maple syrup urine disease, congenital adrenal hyperplasia, and additional hemoglobinopathies are potentials for future inclusion. These disorders are detectable in the pre-symptomatic period, and have effective interventions. They are therefore suitable for the expansion of the NBS programme as diagnostic capacity, treatment infrastructure, and public health resources improve.

To establish a sustainable NBS programme, the workshop participants proposed an integrated implementation strategy that would encompass several key components:

1. Integration of NBS into Maternal and Child Health Services: A key strategy for NBS is its integration into routine maternal and child health services to streamline service delivery, enhance efficiency, and improve continuity of care.
2. Technical Infrastructure and Laboratory Capacity: The implementation of NBS will hinge on a robust technical infrastructure to deploy cost-effective and reliable laboratory solutions to screen CH and SCD from DBS samples. Laboratories with high diagnostic standards will be developed alongside establishing a national database to track screening outcomes, manage follow-up care, and monitor programme performance
3. Public–Private Partnerships (PPPs): This will address the significant resource and infrastructure deficits. The consortiums recommended collaborations with industry partners that can provide the necessary financial and technical support to upgrade laboratory facilities and procurement of equipment. The model will also facilitate training programmes for healthcare workers, ensuring a consistent supply of skilled professionals for screening and follow-up care. Innovative funding models that combine government budgets with private sector investment will also ensure the long-term sustainability of the screening programme.
4. Legislative and Policy Framework: A framework critical for the successful adoption of newborn screening would include collaboration between the government and healthcare experts. Both groups would formulate a national policy that mandates newborn screening as part of the standard of care. This policy would align with global standards and recommendations.

The participants unanimously agreed that achieving a successful national NBS programme will require concerted efforts in policy advocacy, capacity building, and public awareness. These will be achieved by:

1. Stakeholders engaging with government officials and legislators to elevate the priority of newborn screening on the national health agenda. This involves presenting evidence-based arguments on the cost-effectiveness and long-term benefits of early detection.
2. Launching advocacy campaigns that highlight the potential of NBS to save lives and reduce healthcare costs in Nigeria to policymakers, healthcare providers, and the public, using the success stories of countries with established programmes.
3. Establishing a national training programme for healthcare workers’ capacity building that would cover all aspects of newborn screening, from sample collection and laboratory processing to genetic counselling and long-term patient management.
4. Creating educational initiatives for the community aimed at raising awareness about the benefits of early screening and dispelling common myths.
5. Regular media briefings, public service announcements, and social media campaigns to disseminate information about the NBS programme to a larger audience.
6. Providing educational resources and support networks for parents of children diagnosed with congenital conditions to ensure that families are well-informed and able to access the interventions.
7. Expanding the scope of diseases screened after the initial programme for SCD and CH is operational. This phased approach will allow for the gradual enhancement of screening services as laboratory capacity and funding improve.

### 2.3. Recommendation Highlights

The Newborn Screening Consortium–Nigeria resolved to:

1. Adopt the World Health Assembly (WHA) resolution on universal newborn screening, ensuring that no newborn is left behind, as endorsed by the Coordinating Minister of Health.
2. Commence a national comprehensive NBS programme beginning with congenital hypothyroidism and sickle cell disease, building laboratory capacity, and having semi-automatic platforms for detecting SH and SCD variants from DBS.
3. Advocate for the integration of newborn screening as a national health priority within Nigeria’s health policy framework.
4. Collaborate with the Federal Government to develop and implement a nationwide integrated NBS policy and implementation plan.
5. Mobilise resources and partnerships to establish and strengthen newborn screening services across all geopolitical zones.
6. Promote continuous education and training for healthcare professionals and public awareness on the importance of early detection and management of newborn conditions.

## 3. Conclusions

Newborn screening is not just a detection test but a comprehensive healthcare system that prevents disabilities and saves lives. The National Workshop on Newborn Screening in Nigeria has raised the urgent national awareness on the need to address the current gaps in neonatal healthcare. The NSC-N remains committed to working collaboratively towards achieving universal newborn screening in Nigeria. The recommendations in this document provide a comprehensive pathway to establish a sustainable, integrated newborn screening programme that is evidence-based and relevant to the Nigerian health system.

### Next Steps

In translating the resolutions into actionable policies and setting coordinated actions aimed at building an equitable, data-driven newborn screening system that gives every child a right to a healthy start in life, the consortium’s immediate next steps included the following.

National Technical Working Group:

A National Technical Working Group will be established to provide strategic oversight, coordinate stakeholders, monitor implementation, and support the sustainability and scale-up of newborn screening (NBS) services.

Policy and Legislative Advocacy:

Engagements with the Federal Ministry of Health and relevant legislative bodies will be undertaken to secure policy endorsement, legislative support, and integration of NBS into national child health frameworks.

Progress Review and International Collaboration:

A six-month follow-up stakeholders’ meeting was planned to review progress on agreed actions. This meeting was held alongside the World Sickle Cell Congress in Nigeria, facilitating progress assessment and knowledge exchange with countries operating established newborn screening programmes.

## Data Availability

No new data were created or analyzed in this study. Data sharing is not applicable to this article.
